# Central University of Kentucky

**Published:** 1887-08

**Authors:** 


### The Central University of Kentucky—Department of Dentistry.

The first annual commencement of the Dental Department of the Central University of Kentucky (Louisville College of Dentistry) was held in connection with that of the Medical Department (Hospital College of Medicine) in Masonic Temple, Lousiville, Kentucky, Tuesday, June 14, 1887, at 8 p. m.

The valedictory address was delivered by Chas. Kelso Runyon of Indiana, and the address to the graduating class by Prof. Jas. Louis Howe. The degree of D.D.S. was conferred upon the following gentlemen by L. H. Blanton, Chancellor: J. C. Blair, Miss.; J. W. Creed, Ky.; W. W. Griffiths, Texas; W. P. Moore, Ill.; E. T. Morgan, Fla.; C. K. Runyon, Ind.; J. C. Steen, Ohio; W. W. Steen, Ky.; J. W. Trainor, Ind.; J. Van Eldren, Ky.

